# Growth differentiation factor 11 induces skeletal muscle atrophy via a STAT3-dependent mechanism in pulmonary arterial hypertension

**DOI:** 10.1186/s13395-022-00292-x

**Published:** 2022-05-06

**Authors:** Guiling Xiang, Kelu Ying, Pan Jiang, Mengping Jia, Yipeng Sun, Shanqun Li, Xiaodan Wu, Shengyu Hao

**Affiliations:** Department of Pulmonary and Critical Care Medicine, Zhongshan Hospital, Fudan University, 180 Fenglin Road, Shanghai, 200032 China

**Keywords:** Pulmonary arterial hypertension, GDF11, Skeletal muscle atrophy, STAT3

## Abstract

Skeletal muscle wasting is a clinically remarkable phenotypic feature of pulmonary arterial hypertension (PAH) that increases the risk of mortality. Growth differentiation factor 11 (GDF11), centrally involved in PAH pathogenesis, has an inhibitory effect on skeletal muscle growth in other conditions. However, whether GDF11 is involved in the pathogenesis of skeletal muscle wasting in PAH remains unknown. We showed that serum GDF11 levels in patients were increased following PAH. Skeletal muscle wasting in the MCT-treated PAH model is accompanied by an increase in circulating GDF11 levels and local catabolic markers (Fbx32, Trim63, Foxo1, and protease activity). In vitro GDF11 activated phosphorylation of STAT3. Antagonizing STAT3, with Stattic, in vitro and in vivo, could partially reverse proteolytic pathways including STAT3/socs3 and iNOS/NO in GDF11-meditated muscle wasting. Our findings demonstrate that GDF11 contributes to muscle wasting and the inhibition of its downstream molecule STAT3 shows promise as a therapeutic intervention by which muscle atrophy may be directly prevented in PAH.

## Introduction

Despite improvement in morbidity and mortality with the emergence of targeted therapies, most PAH patients remain symptomatic. Persistent exercise intolerance also significantly impaired their quality of life [1]. Although exercise intolerance was traditionally considered to arise from cardiac dysfunction, some previous studies have suggested that skeletal muscle dysfunction may also contribute to the exercise limitation in patients with PAH [2, 3]. Pulmonary rehabilitation could improve exercise capacity [4]; however, it is necessary to develop anabolic drugs which might improve patients’ outcomes.

Skeletal muscle atrophy represents an imbalance between protein synthesis and breakdown [5]. In experimental monocrotaline (MCT)-induced PAH in rats, morphological and functional skeletal muscle changes were related with impaired exercise capacity, including smaller muscle fiber cross-sectional area (CSA), decrease in contractile function and upregulation of pro-atrophic ubiquitin ligases (Fbx32, Trim63) which contributed to proteolysis, compared to control rats [6, 7]. The local markers of proteolysis, such as myostatin (MSTN) and protease activity also increased in the MCT-induced PAH model [8]. Furthermore, Fbx32 and Trim63 were overexpression in the quadriceps of patients with PAH, while insulin-like growth factor 1 (IGF-1) pathway and kinase B (AKT) contributed to protein synthesis, were downregulated [5, 9].

Several catabolic myokines in transforming growth factor β (TGFβ) family, including MSTN, growth differentiation factor 11 (GDF11), and growth differentiation factor 15 (GDF15), are associated with muscle wasting [10, 11], even in PAH patients [12]. The TGFβ proteins have been involved in PAH pathogenesis [13, 14]. Recently, Yu et al. [15] provided critical insights that GDF11 were activated in the endothelial region of pulmonary arteries and involved in pulmonary artery endothelial cells (PAEC) proliferation and angiogenesis.

In addition to loss of appetite, higher GDF11 levels cause loss of skeletal muscle which is consistent with the report mentioned earlier that GDF11 is a more potent ligand for Alk than is MSTN, a potent muscle-inhibiting cytokine [16, 17]. Schafer et al. reported that when high GDF11 levels were observed in older patients with aortic stenosis, the prevalence of frailty and prior cardiac conditions were increased [18]. The finding makes it of interest to investigate whether high GDF11 levels in humans are sufficient to contribute to any symptoms of frailty such as muscle atrophy.

The downstream signaling pathways of GDF11 in skeletal muscle remain to be elucidated. Furthermore, multiple potential targets have been implicated in GDF11 or MSTN which is closely related to GDF11, including NFκB, STAT3, ERK, and Smad pathways [19–21]. It is of importance to identify and target the downstream signaling pathways of GDF11 in the muscle which is a potential method to develop targeted drugs for muscle wasting in patients with PAH even in other conditions.

In the current study, we therefore aim to identify the systemic or local GDF11 levels in PAH patients and MCT-induced PAH model, to identify key signaling pathways through which GDF11 acts in muscle with a view to developing novel therapies.

## Materials and methods

### Reagents and antibodies

Antibodies against p-STAT3 (Tyr705) (#9145), STAT3 (#9139), p-Smad2 (Ser465/467)/Smad3 (Ser423/425) (#8828), and Smad2/3 (#8685) were purchased from Cell Signaling Technology (Beverly, MA); antibodies against GDF11 (sc-81952) and SOCS3 (sc-51699) were purchased from Santa Cruz Biotechnology (Santa Cruz, CA); antibodies against Fbx32 (ab168372), CD31 (ab28364), and wheat germ agglutinin (ab178444) were purchased from Abcam (Cambridge, MA); recombinant GDF11 protein (1958-GD) were purchased from R&D Systems (Minneapolis, MN); antibodies against Trim63 (55456-1-AP), ubiquitin (10201-2-AP), iNOS (18985-1-AP), and GAPDH (HRP-60004) were purchased from ProteinTech group (Rosemont, IL); and antibodies against FoxO1 (BS1746) were purchased from bioworld (Bloomington, MN). Crotaline (C2401) were purchased from Sigma-Aldrich (St. Louis, MO). Stattic (S7024) were purchased from Selleck (TX, USA). Human GDF11 ELISA Kit (QC-GDF11-Hu), rat GDF11 ELISA Kit (QC-GDF11-Ra), and mouse GDF11 ELISA Kit (QC-GDF11-Mu) were purchased from QCHENG BIO (Shanghai, China). Human MSTN ELISA Kit (E-EL-H1437c) and human Activin A ELISA Kit (E-EL-H0003c) were purchased from Elabscience Biotechnology (Wuhan, China). MG-132 was purchased from Tocris Bioscience (Minneapolis, MN).

### Human study

Ethical approval was granted by the Fudan University Zhongshan Hospital (Approval number: B2018-184R). In 8 patients with PAH (age 51–82 years, 25% females) recruited from the Zhongshan Hospital, we had taken serum for the analyses of circulating GDF11, MSTN, and Activin A. Age/sex-matched blood donors served in this study as healthy controls.

### Cell cultures

C2C12 cells (Cell Bank of Shanghai Branch, Chinese Academy of Sciences) were grown in Dulbecco’s Modified Eagle Medium (DMEM, Gibco, MA, USA) supplemented with 10% fetal bovine serum (FBS, Bioind, Israel) plus 1% penicillin/streptomycin (Gibco, MA, USA). To induce myotube formation, C2C12 cells were grown to 100% confluency, then the medium was switched to DMEM containing 2% horse serum (Gibco, MA, USA) for up to 5 days. The myotubes size was determined by measuring the average fiber widths of long fibers by Image-J software. Myotubes were measured in 20 randomly selected fields per condition for each experiment.

Mouse PAECs were isolated and cultured in Endothelial Cell Medium (Sciencell, USA) containing 8% FBS as described previously [22]. All cells were cultured at 37°C in 5% CO_2_ and 95% relative humidity. Myotubes were treated for a range of doses with control or GDF11 (0 ~ 100ng/mL) in the presence or absence of Stattic (5 μM) or MG-132 (10 μM) and siGDF11 or control siRNA (pLKO.1). PAECs were grown to 30–50% confluence and then transfected with siRNA (GDF11 or control) using Lipofectamine 2000 (Invitrogen, CA, USA). After 6 h transfection, cells were cultured in a serum-containing medium for a period of 48 h for protein knockdown. All siRNA was synthesized by GenePharma (Shanghai, China).

### Conditioned medium

PAECs (5×10^6^ cells) were seeded in 10 mm dishes and grown to 80% confluence. Then, cells were cultured in hypoxia (1% O_2_) or normoxia respectively for 24 h. Subsequently, the supernatant as conditioned medium (CM) from PAECs was collected and centrifuged at 450×*g* for 5 min, 4 °C; the CM was diluted with fresh differentiation media. Myotubes were treated with the diluted CM for 48 h.

### Animals

Experimental protocols were approved by the Ethics on Animal Care and Treatment Committee of Fudan University Zhongshan Hospital and conducted according to the National Institutes of Health Guide for the Care and Use of Laboratory Animals. Adult male Sprague-Dawley rats, 6-7 weeks old, were purchased from the JSJ Laboratory Animal Co. Ltd (Shanghai, China). The rat model of PAH was established as described previously [23]. Briefly, rats received subcutaneous injections of MCT (40 mg/kg) or vehicle control.

In the STAT3 inhibition study, 36 rats were treated with MCT or vehicle control as above. After 14 days, 8 of the MCT rats and 8 of the rats in the control groups received daily intraperitoneal injections of Stattic (1.25 mg/kg, dissolved in 10% DMSO/90% PBS) for 14 days. The rest of the rats were treated with diluent (10% DMSO/90% PBS) for 14 days. Body weights were measured daily. Food intake was monitored on a per cage basis from the beginning of Stattic-treated.

All rats were killed at 4 weeks humanely, shortly after haemodynamic assessment which was evaluated by echocardiography and invasive right ventricular (RV) pressure measurements [24]. The weights of muscles were then determined. Subsequently, soleus, extensor digitorum longus, tibialis anterior and gastrocnemius muscles were dissected, weighed, quickly frozen in liquid nitrogen, and stored at 80 °C for further analysis.

### Immunohistochemistry and immunofluorescence staining

Sections of lung tissue and gastrocnemius muscles were embedded in paraffin. Sections were blocked in donkey serum followed by incubation with GDF11 antibody or CD31 antibody for lung tissue, incubation with wheat germ agglutinin (WGA) for gastrocnemius muscles at 4°C overnight. Images were photographed with a 40× objective lens on an upright Olympus BX53F microscope. The CSA of myofiber determined from a minimum of 250 myofibers per group were analyzed by Image-J software.

### Western blot and ELISA

Samples from tissue and myotubes were heated to 95°C for 10 min. Then, samples at various concentrations were loaded in SDS-PAGE gels and proteins were detected via specific antibodies. Blots were detected on a Tanon-5500 Imaging System (Shanghai, China). The intensity of the bands was measured via densitometry using ImageJ software.

Serum from human, rat, or CM from the supernatant of PAEC were measured for GDF11, MSTN, or Activin A production using the ELISA Kit as manufacturer’s instructions.

### Luciferase reporter assay

Myotubes were transfected with luciferase reporter plasmids (NF-Κb, STAT3, Smad, ERK) with Lipofectamine 2000. Luciferase activity was measured with a luciferase assay kit (Promega, Madison, WI) according to the manufacturer’s instructions.

### Detection of nitric oxide (NO)

The detection of NO in the medium was conducted using Griess reagent as described previously [25]. The OD value was measured with a microplate reader at 543 nm.

### Statistical analysis

The data are presented as means ± SEM. Student’s *t* test was used when continuous variables were compared, followed by ANOVA with the appropriate post hoc test for multiple comparisons. Statistical analyses were performed using Prism 6 (GraphPad Software Inc) software. *P* values less than 0.05 were considered significant (*, *p* < 0.05; **, *p* < 0.01; ***, *p* < 0.001; NS means not significant, throughout the paper).

## Results

### Increased serum concentrations of GDF11 in PAH patients

**Fig. 1:**
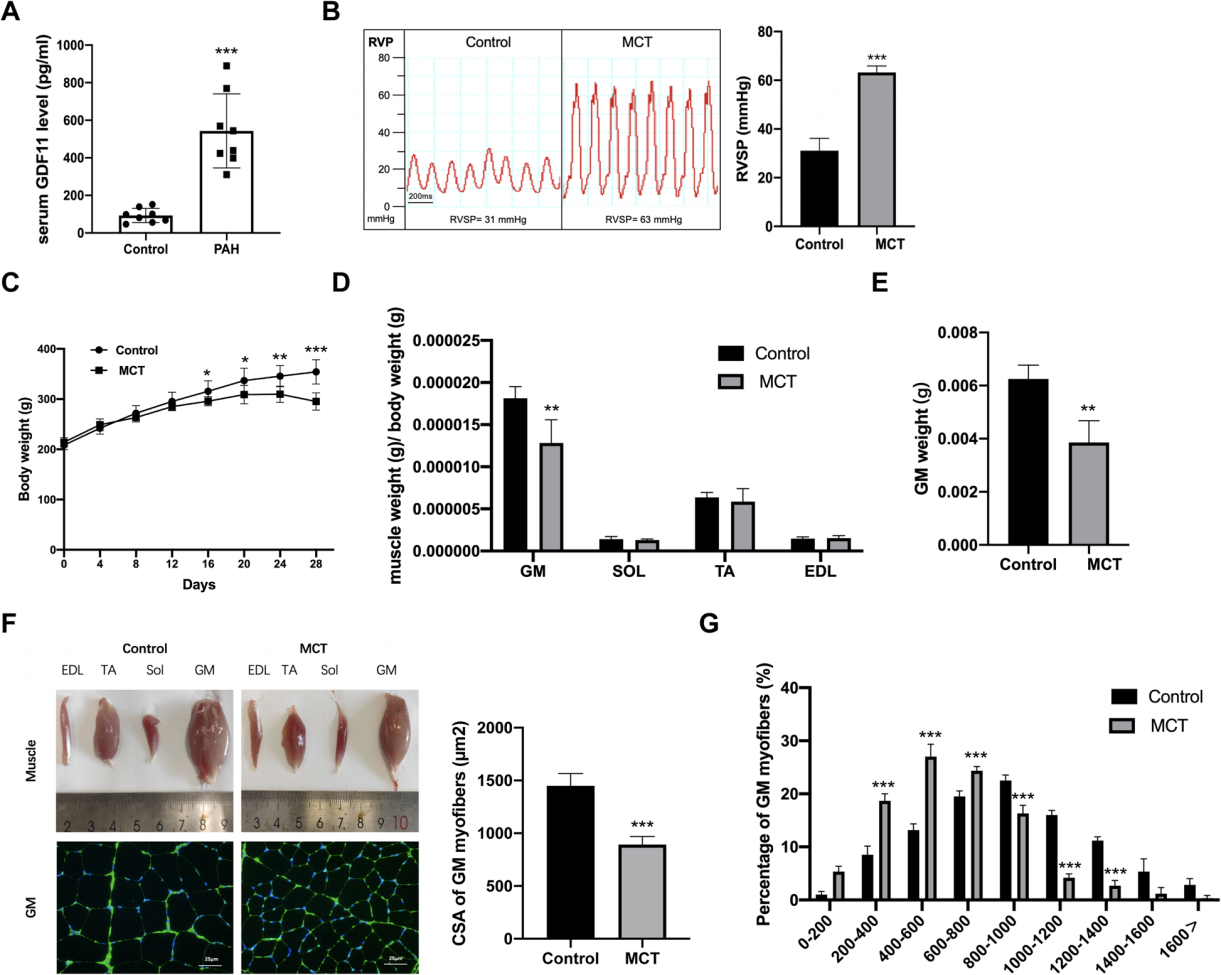
MCT-induced PAH caused body weight loss and muscle atrophy. **A** Circulating GDF11 levels in PAH patients and health control (*n*=8). **B** RVSP of rats 4 weeks after MCT-treated, **C** body weight, and **D** the weight of gastrocnemius muscle, soleus muscle, tibialis anterior, and Extensor Digitorum Longus normalized per body weight (BW). **E** The weight of gastrocnemius muscle. **F** Representative images of EDL, TA, Sol, GM, and the cross-sectional areas of approximately 250 myofibers per group were determined. Scale bar represents 25 μm. **G** The distribution of myofiber cross-sectional area. *n* = 8 rats/group. Data are shown as mean ± SEM. Versus vehicle control, *P < 0.05, **P < 0.01, ***P < 0.001

As seen in Fig. 1A, serum concentration of GDF11 increased significantly in PAH patients (543.1 ± 197.2 pg/ml) compared to healthy controls (92.9 ± 37.8 pg/ml). We also detected the serum concentration of MSTN and Activin A, the molecules closely related to GDF11, and transduced SMAD2/3 activation and downstream transcriptional responses [14]. Although the expression of MSTN and Activin A was higher in PAH patients, only circulating GDF11 levels in PAH patients had the most significantly increased. Therefore, we supposed that GDF11 may play a key role in patients with PAH.

### MCT induces a skeletal muscle wasting phenotype in a PAH rat model

All MCT rats developed PAH as indicated by the increase in RVSP (Fig. 1B). Loss of weight in MCT rats was observed 4 weeks after MCT injection (17% reduction; Fig. 1C). Although the MCT rat tibialis anterior (TA), gastrocnemius muscle (GM), and soleus (SOL) exhibited muscle loss, only the GM atrophy index (muscle weight/total weight) was reduced which was accompanied by a significant decrease of CSA (Fig. 1D–G). Therefore, further research was limited to the GM.

### GDF11 levels are accumulated in serum and lung in MCT rats

**Fig. 2:**
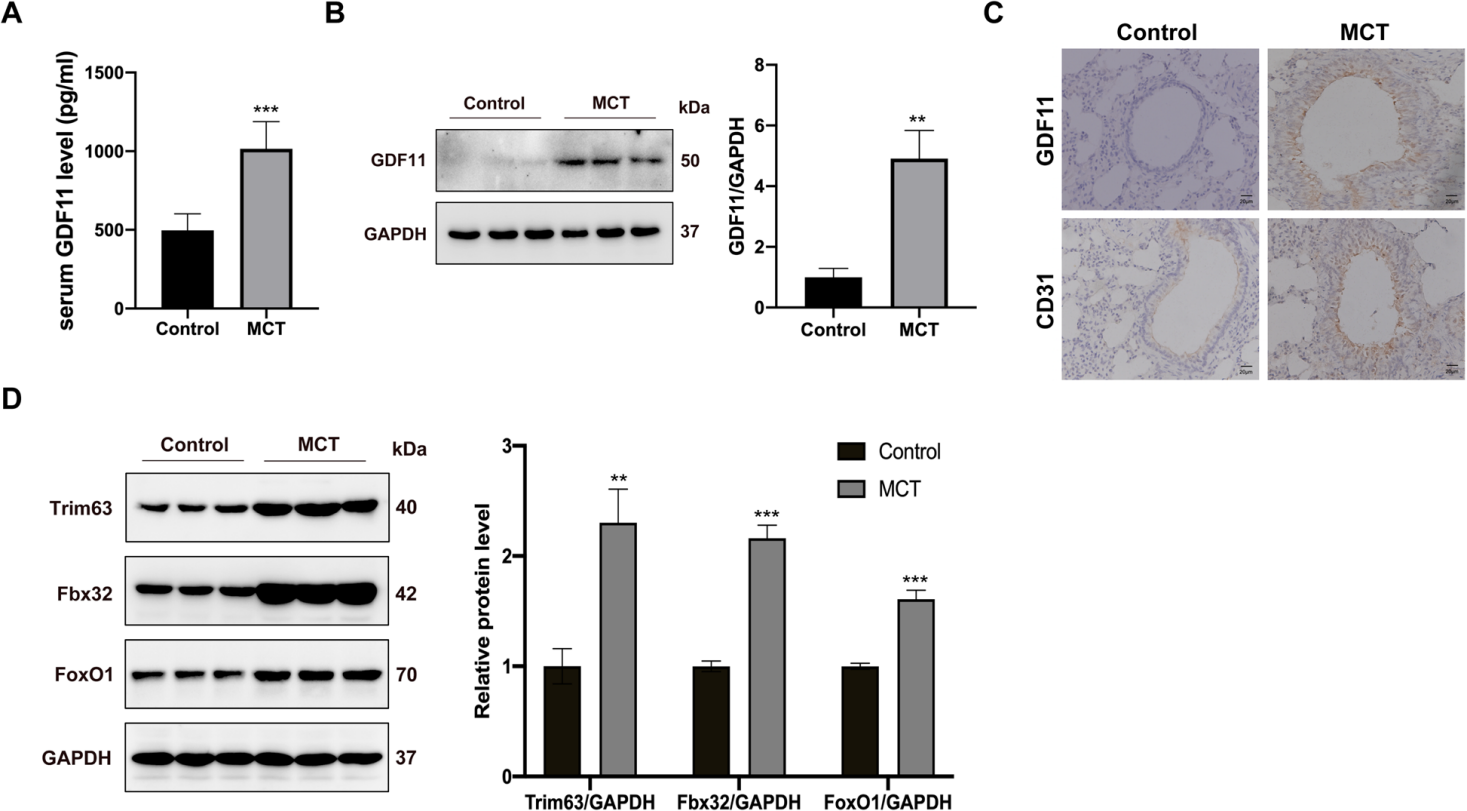
GDF11 levels are accumulated in serum and lung in MCT rats. **A** GDF11 was measured by ELISA in the serum from rats. **B** GDF11 expression in lung was detected by Western blot, and GAPDH served as a loading control. **C** Representative immunohistochemistry of lung sections showing pulmonary arteries stained for GDF11 or CD31 in rats, scale bar is 20 μm. **D** Western blot analysis of trim63, fbx32, and foxo1 was assessed by Western blot, and protein expression levels were quantified by densitometry. Data are shown as mean ± SEM. Versus vehicle control, *P < 0.05, **P < 0.01, ***P < 0.001, *n* = 8 rats/group

Serum GDF11 levels were raised in MCT rats when compared with controls (Fig. 2A). GDF11 protein levels were also elevated remarkably in the lung of MCT rats compared with controls (Fig. 2B). In the MCT model, immunohistochemical staining for GDF11 showed that the expression of GDF11 is higher in the pulmonary arteries which are concentrated in the endothelial cells in MCT-treated rats than in the control group (Fig. 2C). In addition, the protein levels of Trim63, Fbx32, and Foxo1, which are important factors in the ubiquitin-proteasome pathway, were raised significantly in GM of MCT rats when compared with controls (Fig. 2D).

### In vitro model of PAH-derived GDF11 is sufficient to induce the myotube atrophy

**Fig. 3:**
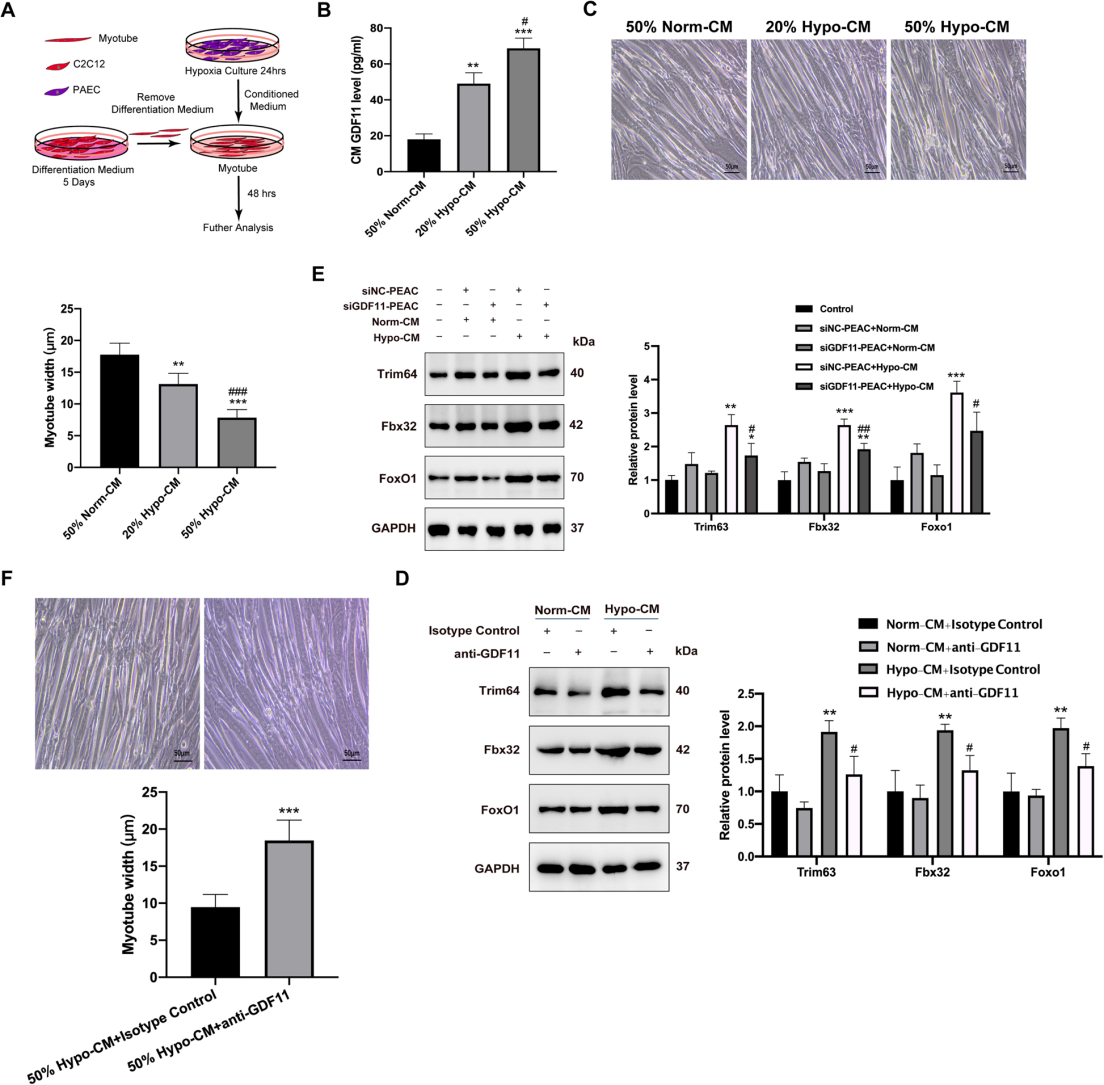
The GDF11 is involved in myotube atrophy induced by CM from PAEC. **A** Schematic drawing depicting the generation of CM by hypoxia-culture of PAEC, then myotube was stimulated with CM for 48 h. **B** Concentrations of GDF11 (pg/mL) in 50% Norm-CM, 20% or 50% Hypo-CM. **C** Bright-field images of C2C12-derived myotubes treated with either 50% Norm-CM, 20% or 50% Hypo-CM from PAEC, and myotube diameter for conditions represented in the panel. Scale bar is 50 μm. **D** Myotubes were transfected with GDF11 siRNA or NC siRNA and 50% Norm-CM or 50% Hypo-CM. Protein levels were examined by immunoblotting. **E** Bright-field images of myotubes treated with 50% Hypo-CM with GDF11 antibody or isotype control, and myotube diameter for conditions represented in the panel. Scale bar is 50μm. **F** Immunoblots of trim63, fbx32, and foxo1 using lysates from myotubes treated with 50% Norm-CM or 50% Hypo-CM with GDF11 antibody or isotype control; and protein expression levels were quantified by densitometry. Values are presented as average ± SEM. Versus vehicle control, *P < 0.05, **P < 0.01, ***P < 0.001; versus Hypo-CM control, ^#^*P* < 0.05, ^##^*P* < 0.01, ^###^*P* < 0.001; *n*=3

To explore the signaling pathways which contribute to muscle wasting in PAH, we utilized a PAEC-induced myotube atrophy model. The myotube was from the differentiation of C2C12. We collected CM from PAEC which was under hypoxia culture for 24h. The myotubes were treated with Hypo-CM (20%, 50%) for 48 h, and then fixed for the measurement of myotube diameter (Fig. 3A). Hypo-CM induced a decrease in myotube diameter which was accompanied by an increase of GDF11 level in Hypo-CM (Fig. 3B, C).

To verify whether Trim63, Fbx32, and Foxo1 was a downstream target of GDF11 in myotubes, GDF11 was silenced with specific siRNA. We found that loss of GDF11 down-regulated Trim63, Fbx32, and Foxo1 expression in Hypo-CM treated myotubes (Fig. 3D).

Next, the myotube diameter was measured in response to anti-GDF11 (neutralizing GDF11 antibody) or isotype control. Notably, anti-GDF11 could alleviate the myotube atrophy induced by 50% Hypo-CM significantly (Fig. 3E). Furthermore, Western blotting of treated myotubes demonstrated inhibition of proteolysis by anti-GDF11 showed as the reduction of Trim63, Fbx32, and Foxo1 levels (Fig. 3F). These results demonstrate that GDF11 released from in vitro model of PAH induce significant myotube atrophy.

### GDF11 acts via STAT3, SOCS3, and iNOS to induce proteolysis in muscle atrophy in vitro

**Fig. 4:**
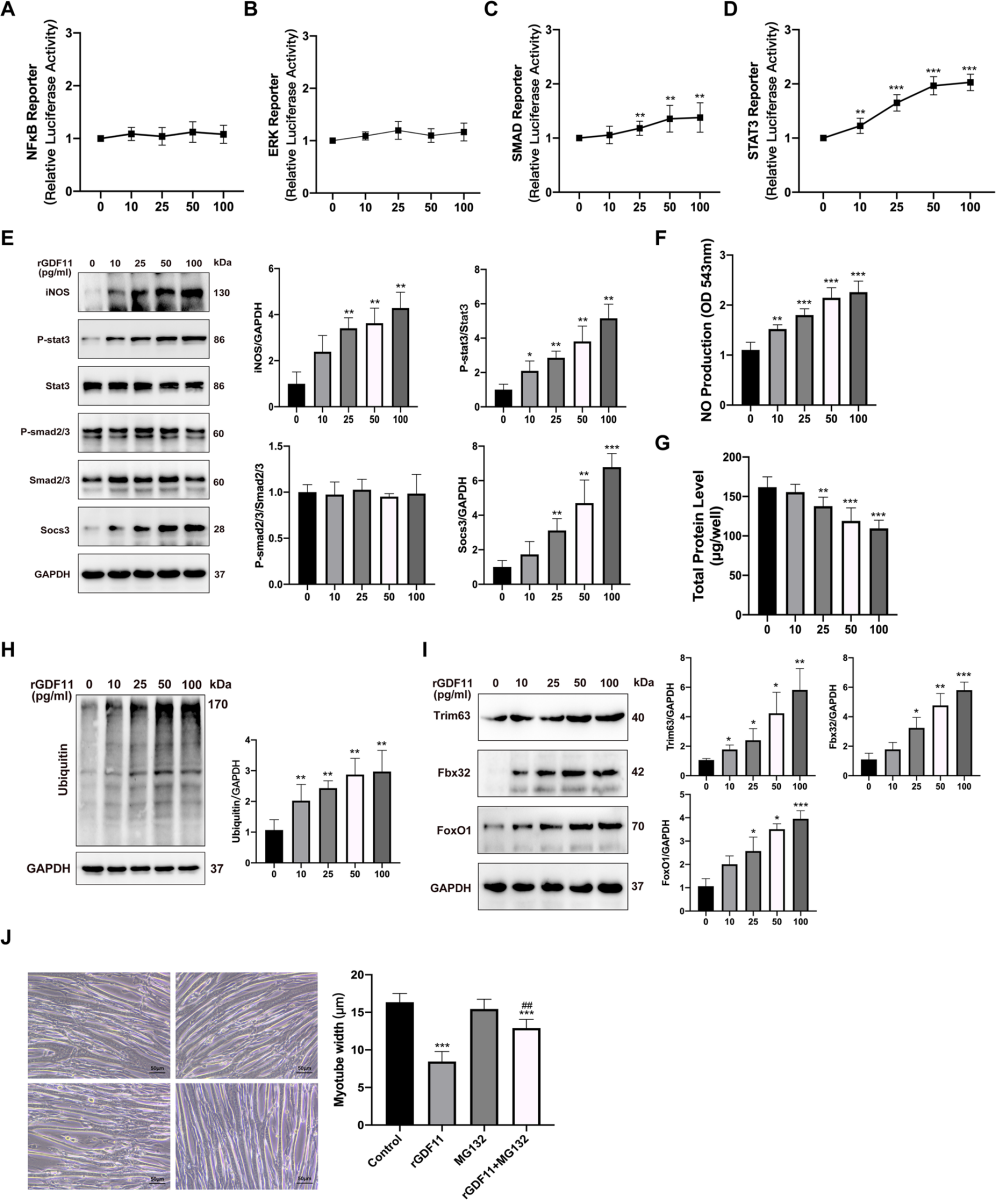
GDF11 acts via STAT3, SOCS3, and iNOS to induce proteolysis in muscle wasting in vitro. **A**–**D** NF-κB, ERK, Smad, or STAT3 dependent luciferase reporters in C2C12 myotubes treated with rGDF11 with the dose ranging from 0 to 100 ng/ml. **E** Representative Western blots of target proteins (iNOS, phosphorylation and total STAT3, phosphorylation and total Smad2/3, socs3) and loading control (GAPDH) from myotubes treated with rGDF11 with the dose ranging from 0 to 100 ng/ml for 48 h. **F** NO levels were measured in supernatant from the myotubes described in the panel. **G** Total protein content of rGDF11-treated myotubes. **H** Representative western blotting images of ubiquitin from myotubes. **I** Protein expression of trim63, fbx32, and foxo1 in myotubes treated with rGDF11 with the dose ranging from 0 to 100 ng/ml. GAPDH was used as an internal control. **J** Bright-field images of myotubes treated with rGDF11, with or without the 26S ribosome inhibitor MG-132 (10 μM) for 48 h; diameter of myotubes for conditions represented in the panel. Scale bar is 50μm. Data presented as mean ± SEM. Versus vehicle control, *P < 0.05, **P < 0.01, ***P < 0.001; versus rGDF11 control, ^#^*P* < 0.05, ^##^*P* < 0.01, ^###^*P* < 0.001; *n*=3

To further investigate intracellular signaling pathways activated in myotube in response to GDF11, we transfected C2C12 with NF-κB, ERK, Smad, or STAT3 dependent luciferase reporter plasmids. After differentiation of myotubes, the myotubes were subsequently treated with rGDF11 for 48 h. We selected these transcription factors for which were potential targets and have been implicated in skeletal muscle atrophy [19–21]. Neither NF-κB nor ERK-dependent reporter activity changes in response to rGDF11 treatment (Fig. 4A, B). However, rGDF11 induced activation of the Smad reporter (Fig. 4C). rGDF11 also significantly increased STAT3 reporter activity in a dose-dependent manner (Fig. 4D). Based on these findings, we next measured the activation of Smad2/3 and STAT3 in myotubes treated with rGDF11. rGDF11 treatment induced a significant increase in phospho/total STAT3 and the effect was concentration-dependent, especially at the dose of 50 and 100 ng/ml (Fig. 4E). However, we had not found a significant increase in phospho/total Smad2/3 levels (Fig. 4E).

We next measured protein levels of iNOS and socs3 since both would be increased in response to activation of STAT3 in atrophic myotubes in other conditions [26, 27]. Levels of iNOS and socs3 were significantly increased at 48h of rGDF11 treatment with a corresponding increase in the production of NO, which was paralleled by an increase in phospho/total STAT3 (Fig. 4E, F), suggesting they were the potential target of STAT3 which was activated by GDF11.

Correspondingly, we detected reduced protein content in myotubes treated with rGDF11 (Fig. 4G). The ubiquitin-proteasome system (UPS) and Foxo1could regulate muscle degradation via the E3 ubiquitin ligases, Trim63 and Fbx32 [28]. In our study, the levels of UPS were increased at 48h of rGDF11 treatment with a corresponding increase in the expression of Trim63, Fbx32, and Foxo1 dose-dependently (Fig. 4H, I). Based on these findings, we suppose that the exposure of myotubes to GDF11 activates STAT3 pathways implicated in skeletal muscle wasting in PAH.

We set out to determine if GDF11 induce myotube wasting via proteolytic effects. A 26S proteasome inhibitor, MG-132, was added to myotubes. We detected that MG-132 prevented GDF11-induced reductions in myotube diameter (Fig. 4J). These findings suggest that GDF11 has direct proteolytic effects on atrophic myotube via STAT3, SOCS3, and iNOS.

### STAT3 inhibition abrogates myotube atrophy treated by GDF11

**Fig. 5:**
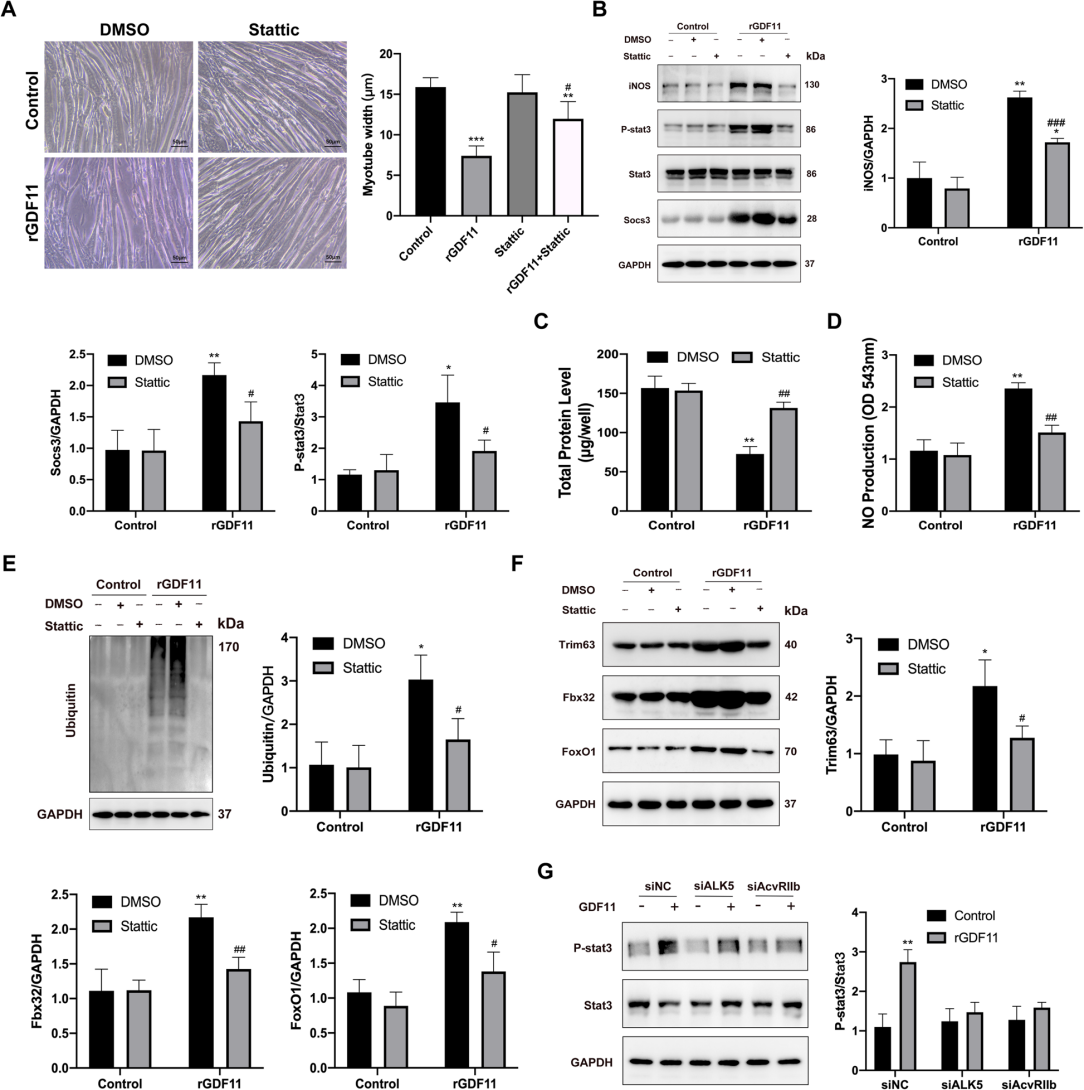
Blocking STAT3 activation with Stattic, a STAT3 inhibitor, prevents GDF11 mediated atrophy in vitro. **A** Bright-field images of myotubes treated with rGDF11 (50ng/ml), with or without STAT3 inhibitor Stattic for 48 h. Scale bars = 50 μm. The fiber widths were measured and calculated (right panel). **B** Myotubes treated with rGDF11 then with Stattic for 48h were used for Western blot analysis with antibodies against iNOS, pY-STAT3, total STAT3, socs3, and GAPDH. **C** Total protein content of rGDF11-treated myotubes, with or without STAT3 inhibitor Stattic for 48 h. **D** NO levels were measured in supernatant from the myotubes described in the panel. **E** Representative western blotting images of ubiquitin from myotubes. **F** Protein expression of trim63, fbx32, and foxo1 in myotubes treated with rGDF11 then with Stattic for 48h. **G** Representative Western blots of phosphorylation and total STAT3 from myotubes treated with rGDF11, siALK5, or AcvRIIb. Data presented as mean ± SEM. Versus vehicle control, *P < 0.05, **P < 0.01, ***P < 0.001; versus rGDF11 control, ^#^*P* < 0.05, ^##^*P* < 0.01, ^###^*P* < 0.001; *n*=3

Results showed that Stattic, a STAT3 inhibitor, reversed myotube atrophy induced by GDF11, as assessed by myotube diameter (Fig. 5A). Next, we observed that Stattic completely inhibited iNOS and socs3 expression in myotube treated with rGDF11 (Fig. 5B). We detected that total protein level inhibition by GDF11 was reversed by Stattic (Fig. 5C). NO production and UPS levels were also dependently decreased by Stattic (Fig. 5D, E). In addition, GDF11-induced expression of Trim63, Fbx32, and Foxo1 protein was drastically inhibited by Stattic (Fig. 5F). These data indicate that Stattic has a therapeutic effect on myotube wasting induced by GDF11.

Canonical signal transduction begins with GDF11 binding to its type II serine/threonine kinase receptor (ACVR2B) which then recruits and activates type I serine/threonine kinase receptors (ALK5) [29]. Results showed that knocked down ALK5 and ACVR2B suppressed the phosphorylation of STAT3. Therefore, we think GDF11 mediated the STAT3 signal which is dependent of ACVR2B/ALK5.

### STAT3 inhibition improves muscle wasting in the MCT-treated rats

**Fig. 6:**
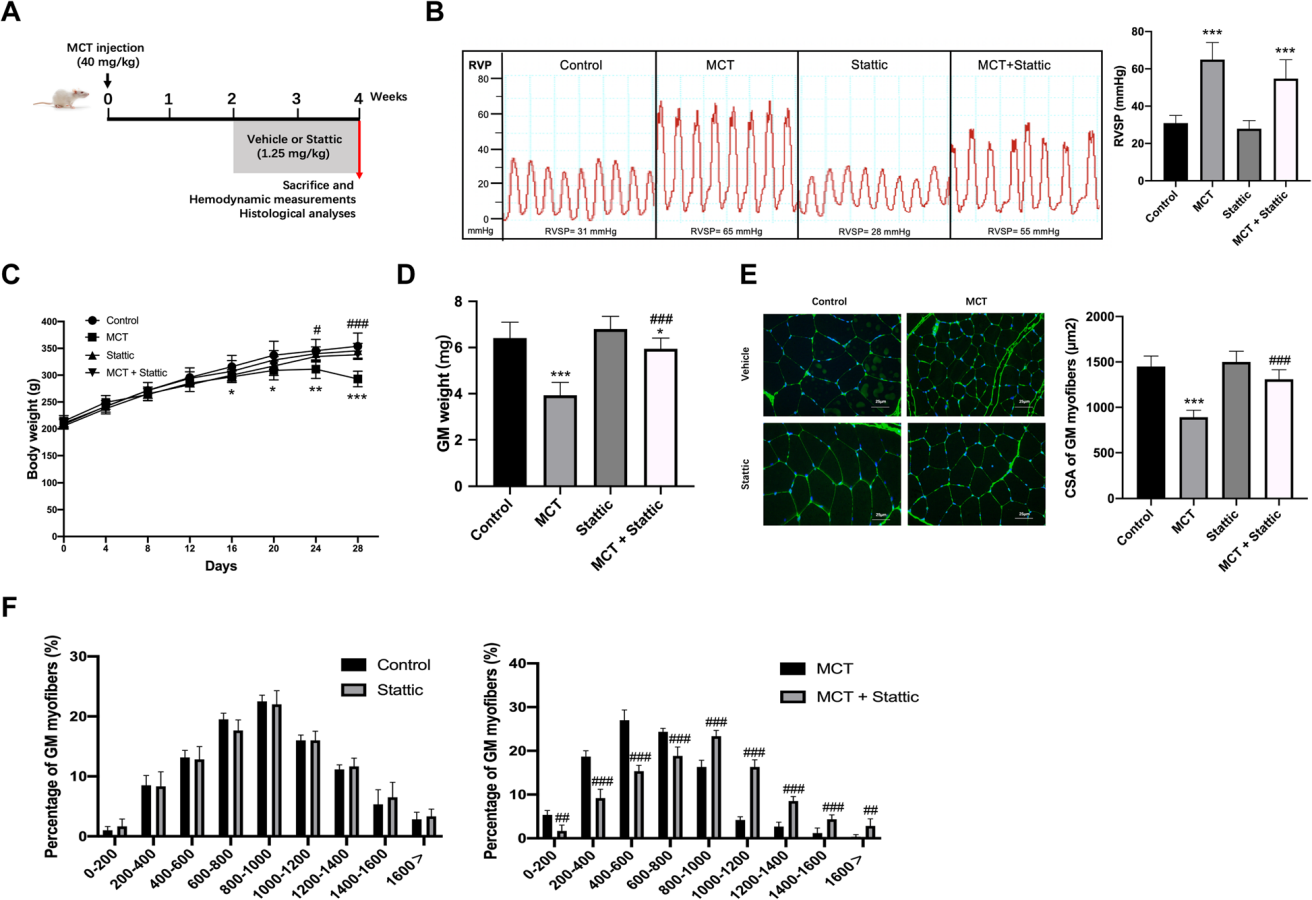
STAT3 inhibition prevents muscle atrophy in the MCT rat. **A** SD rats were treated with MCT or saline on day 1, with or without intraperitoneal injection of Stattic daily for 2 weeks since day 14. The effects of Stattic on the main features of PAH model were examined, including **B** RVSP, **C** body weight, and **D** gastrocnemius muscles mass. **E** Cross-sections cut from the gastrocnemius muscle and stained with wheat germ agglutinin (blue). Scale bar is 25μm, *n*=3. **F** The cross-sectional areas of approximately 250 myofibers per group were determined and the distribution of myofiber cross-sectional area. Data are shown as mean ± SEM. Versus vehicle control, *P < 0.05, **P < 0.01, ***P < 0.001; versus MCT control, ^#^*P* < 0.05, ^##^*P* < 0.01, ^###^*P* < 0.001; *n*=8 rats

We next evaluated the therapeutic effect of STAT3 inhibition in an MCT-treated model of PAH (Fig. 6A). STAT3 inhibition had no significant effect on RVSP (Fig. 6B).

Furthermore, Stattic administration distinctly improved chief features of muscle wasting, with significant prevention of the loss of body weight, GM weight with a corresponding prevention of the decrease of CSA (Fig. 6C–F).

STAT3 is a regulator of leptin signaling, which can affect food intake. However, MCT-treated rats in our study ate significantly less than their control counterparts (354g vs 422g, respectively). There was no difference in average food intake per animal between the MCT and MCT/Stattic-treated rats (354g vs 357g, respectively). This, along with the above data, suggests that STAT3, as a down-stream mediator of GDF-11, has a pro-atrophic effect on the skeletal muscle that is independent of its role as an appetite suppressant.

### STAT3 inhibition improves muscle wasting through inhibition proteolysis

**Fig. 7:**
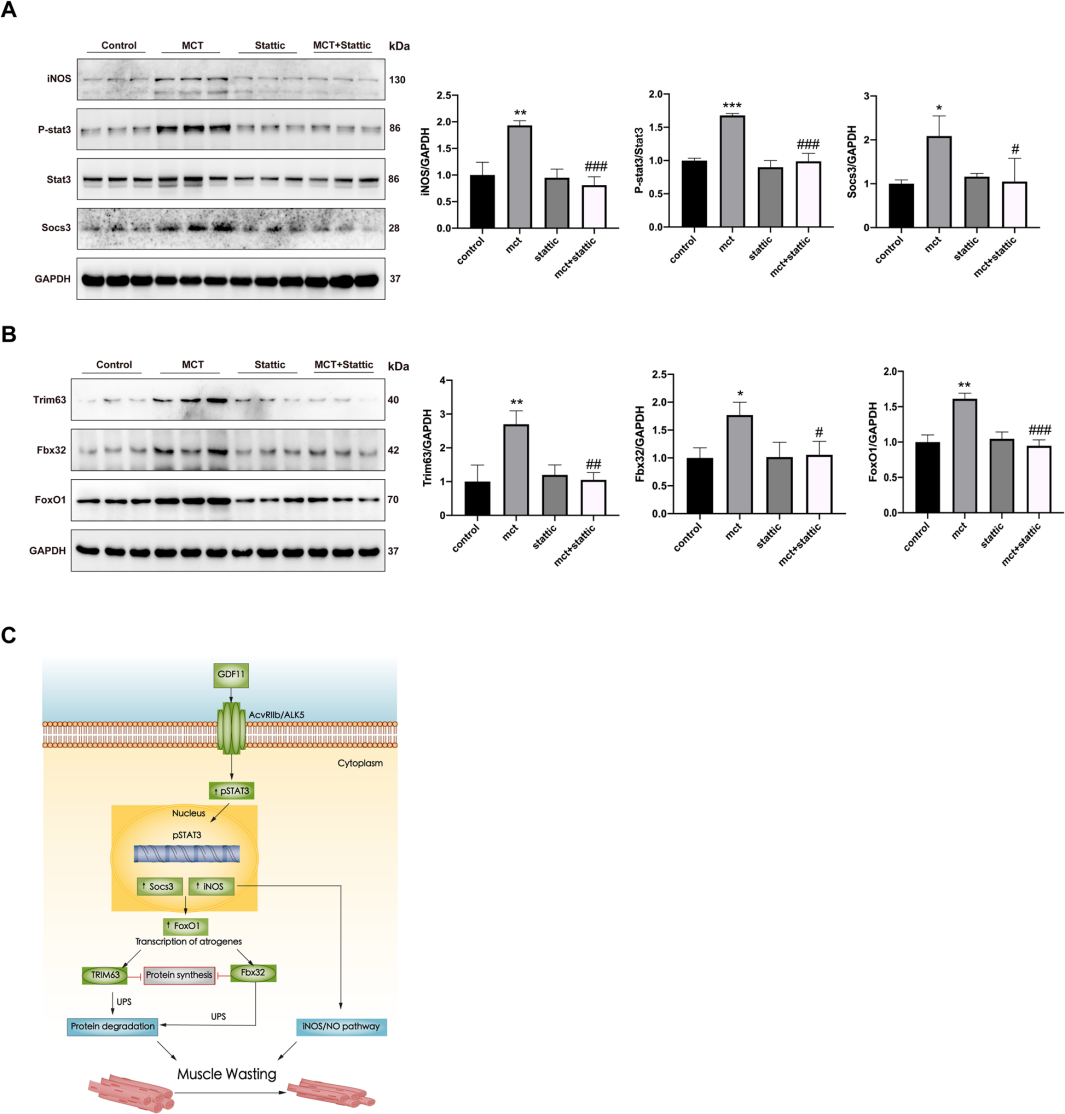
Pathway of STAT3 inhibition in improvement muscle atrophy in the PAH model. **A**, **B** The expression of indicated proteins in gastrocnemius muscles was detected by western blot. The band intensities were quantified and total STAT3 or GAPDH was used as control. **C** Model depicting how STAT3 promotes GDF11-induced muscle wasting. The GDF11 binds to ACVR2B/ALK5 and then activates STAT3 via phosphorylation. Following p-STAT3 translocates to the nucleus and upregulates the expression of iNOS and socs3, leading to the activation of the ubiquitin-proteasome pathway and iNOS/NO pathway, which in turn promotes muscle wasting. Data are shown as mean ± SEM. Versus vehicle control, *P < 0.05, **P < 0.01, ***P < 0.001; versus MCT control, ^#^*P* < 0.05, ^##^*P* < 0.01, ^###^*P* < 0.001; *n*=3 rats

Results showed that Stattic specifically suppressed the phosphorylation of STAT3 and expression of its down-stream target gene iNOS and socs3 in GM of MCT-treated rats (Fig. 7A). Consistent with cellular data, Stattic administration also inhibited the expression of Trim63, Fbx32, and Foxo1 in gastrocnemius muscles of MCT-treated rats as shown by western-blot (Fig. 7B), which further confirmed that STAT3 inhibition improves muscle wasting through inhibition proteolysis.

## Discussion

The current study offers new insights into the molecular mechanisms contributing to PAH-associated skeletal muscle wasting. Our finding shows that skeletal muscle wasting in the MCT-treated PAH model is accompanied by an increase in circulating GDF11 levels and local catabolic markers (Fbx32, Trim63, Foxo1, and protease activity). While our evidence supports that proteolytic pathways including STAT3/socs3 and iNOS/NO in GDF11 meditated muscle wasting, could be partially reversed by the STAT3 inhibitor static (Fig. 7C).

Previous studies have identified that as an activin-related TGF-β protein, circulating MSTN and GDF11 were increased which contributed to MCT-related catabolic phenotype, but the role of the TGFβ family in muscle wasting in PAH is not fully elucidated [8, 12]. As is the case in aging [30], and cancer [31], our data also implicate that skeletal muscle wasting in PAH may also be a phenotype driven by GDF11. Studies in prostate tumor have contented a unidirectional movement of GDF11 protein from tumor to circulation to muscle. In addition, anti-GDF11 antibody has been shown to be able to block muscle loss in tumor-bearing mice confirming its endocrine effects [31]. Our findings also suggest that GDF11 enhances the muscle wasting phenotype in an endocrine manner and pulmonary vascular endothelium could be the potential source for abnormal high serum levels of GDF11.

The balance of anabolic and catabolic processes is of great importance to keep muscle homeostasis, muscle wasting tilts the equilibrium toward catabolism. Muscle wasting is in part due to excessive protein turnover, which could be attributed to proteasome-dependent protein degradation [32]. We show that GDF11 contribute to high levels of ubiquitin ligases Fbx32, Trim63, and Foxo1 in myotubes in culture. Our findings add weight to the argument that GDF11 contributes to muscle wasting through proteolysis. One of the effective ways to prevent muscle atrophy is inhibiting protein degradation signal pathway, so that excess catabolism could be suppressed.

To investigate the mechanisms whereby GDF11 induces proteolysis, we explored the effect of GDF11 on phosphorylation of STAT3, NF-κB, ERK, and SMAD, four crucial protein degradation signal pathways which are implicated in skeletal muscle atrophy [19–21, 33]. Results showed that GDF11 only stimulated the phosphorylation of STAT3, which made us mainly focus on STAT3 pathways for further investigations. Previous studies have identified that the activation of STAT3 is known to stimulate the expression of E3 ubiquitin ligases Fbx32 and Trim63, resulting in muscle wasting, but the role of the STAT3 in muscle wasting of PAH has not been widely analyzed [34]. In our study, the STAT3 pathway may play a crucial role in course of muscle atrophy induced by GDF11 and its inhibition rescues GDF11 mediated muscle wasting through an inhibition in scos3 and iNOS followed by reduction of pro-proteolytic genes.

STAT3 is important for several signaling pathways driving muscle wasting such as C/EBP, MSTN, and IL- 6 [21, 35]. Clinical trials of drugs aiming to block these factors acquired limited success which targeted at the muscle [36]. Stattic has been used to antagonize STAT3 in various disease models including osteoarthritis [37] and Breast Cancer [38]. STAT3 inhibitors may be more appropriate than other drugs which are more specific at antagonizing muscle atrophy. They have the potential to inhibit these synergistic muscle-wasting pathways [39].

STAT3 inhibition has been shown to improve experimental PAH in several previous studies [40, 41]. However, in our study, treatment of STAT3 inhibitor has decreased RVSP in the PAH rat model, without significant changes, which could be explained by severe PAH with the cachexia model used in our study. While our work and others demonstrates STAT3 as a key intermediator for muscle atrophy, the mechanisms by which STAT3 induces muscle wasting to remain elusive. As a transcription factor, STAT3 is known to regulate the expression of target genes, and which of these genes contribute to MCT-induced muscle wasting is unknown. STAT3 activated by cytokines stimulates the expression of SOCSs, which in turn stimulates UPS-mediated degradation of IRS-1 in angiotensin II-induced muscle wasting, leading to suppression of insulin/IGF-1 signaling [42]. Impaired insulin/IGF-1 signaling is closely related to activation of protein degradation [42]. Here, we identify socs3 as a downstream target of STAT3-mediated muscle wasting in PAH. As a key mediator of cytokine-driven muscle wasting, iNOS is contributed to IFNγ/ TNFα-induced muscle atrophy [26]. Our data suggests that iNOS is also involved in MCT-treated muscle wasting which is mediated by STAT3.

Our findings have potential influence outside PAH which adds more evidence implicating that GDF11 and its downstream signaling molecule STAT3 may be a potential candidate for therapeutic intervention aimed at improving muscle mass and even exercise tolerance across a wide range of conditions.

Previous study indicated that GM starts to deteriorate earlier and atrophies at a faster pace than soleus [43]. In our study, only the GM atrophy index was reduced significantly. And we measured receptor expression in muscles. Results showed that the expression of ACVR2B and ALK5 was increased significantly in the GM of MCT treated rats compared to the other muscles of MCT treated rats (not shown). The levels of p-STAT3 increased in the GM compared to the other muscles in MCT-treated rats. So, we think maybe the expression level of ACVR2B/ALK5 was increased in GM, in turn, lead to the increase of levels of p-STAT3. Of course, the question needs further study.

There are some limitations in this study. Firstly, the other sources of GDF11 secretion outside the lung and muscle have not been excluded. Secondly, functional capacity has not been conducted throughout our study which could add weight to our argument. Thirdly, we don't have investigated muscle samples of PAH patients which could add weight to our evidence. Future work showing that lower GDF11 levels are associated with better improved muscle mass in the PAH animal model would further strengthen our argument.

## Conclusion

Our study suggests that the GDF11 plays a key role in the development of muscle wasting in MCT-treated PAH. GDF11’s effects on muscle could be mediated by the activation of STAT3 proteolysis. Proteasome-dependent protein degradation was identified as signs of skeletal muscle wasting in PAH. Socs3 and iNOS are also involved in MCT-treated muscle wasting which is mediated by STAT3. Inhibition of both STAT3 and 26S ribosomal protein units by a specific inhibitor could rescue GDF11-induced atrophy, making STAT3 inhibition a potential target to prevent muscle wasting in PAH.

## Data Availability

All datasets used or analyzed during the current study are available from the corresponding author on reasonable request.

## Abbreviations

MCT: Monocrotaline
CSA: Cross-sectional area
IGF-1: Insulin-like growth factor 1
TGFβ: Transforming growth factor β
GDF11: Growth differentiation factor 11
GDF15: Growth differentiation factor 15
PAECs: Pulmonary artery endothelial cells
CM: Conditioned medium
RV: Right ventricular
WGA: Wheat germ agglutinin
NO: Nitric oxide
TA: Tibialis anterior
GM: Gastrocnemius muscle
SOL: Soleus
BW: Body weight
PAH: Pulmonary arterial hypertension
